# Supplementary material for: Molecular Origin of Blood‐Based Infrared Spectroscopic Fingerprints
Source: Angew Chem Int Ed Engl. 2021 May 26;60(31):17060–9. doi: 10.1002/anie.202103272 (PMC8361728; doi:10.1002/anie.202103272)
Supplement: Supplementary file 1 — Supplementary [file ANIE-60-17060-s001.pdf]

Angewandte Chemie
Eine Zeitschrift der Gesellschaft Deutscher Chemiker

Supporting Information

Molecular Origin of Blood-Based Infrared Spectroscopic Fingerprints**

Liudmila Voronina, Cristina Leonardo, Johannes B. Mueller-Reif, Philipp E. Geyer, Marinus Huber, Michael Trubetskov, Kosmas V. Kepesidis, Jürgen Behr, Matthias Mann, Ferenc Krausz, and Mihaela Žigman**

anie_202103272_sm_miscellaneous_information.pdf

Table S1. An overview of the most abundant proteins in human blood serum. The first three columns contain the names of the proteins ordered by their average concentration, typical levels and the average LFQ values measured for the healthy individuals. It is apparent that the LFQ values are not proportional to the levels in mg/dL, as expected.

The last three columns characterize the fractionation protocol using proteomic measurement of full plasma of twenty individuals and the HSA-depleted fraction of the same samples. All the cases where the depletion is significant are highlighted in bold.

Protein	Reference range in human blood serum, mg/dL	Average LFQ value in full serum of 31 healthy individuals	Percentage in HSA-depleted fraction compared to full plasma	Average concentration in HSA-enriched fraction, mg/dL	Share in HSA-enriched fraction, %
human serum albumin (ALB)	3300-5230 [1]	6.66E+11	**15±4**	3625	93.9
total immunoglobulin G (IGHG1, IGHG2, IGHG3, IGHG4)	614-1295 [2]	1.33E+11	94±35	-	-
immunoglobulin A (IGHA1)	81-591 [3]	3.05E+10	92±50	-	-
alpha-2-macroglobilin (A2M)	194-445 [4]	1.19E+11	136±55	-	-
transferrin (TF)	163-369 [1]	9.55E+10	100±33	-	-
apolipoprotein A1 (APOA1)	119-240 [2]	1.71E+11	92±26	-	-
haptoglobin (HP)	43-304 [5]	5.28E+10	**18±14**	142	3.7
immunoglobulin M (IGHM)	40-302 [3]	3.12E+10	154±139	-	-
alpha-1-antitrypsin (SERPINA1)	85-213 [5]	8.85E+10	120±39	-	-
complement component C3 (C3)	86-184 [2]	1.0E+11	143±36	-	-
Apolipoprotein B (APOB)	52-163 [2]	8.76E+10	160±36	-	-
Alpha-1-acid glycoprotein 1 (ORM1)	51-154 [5]	1.27E+10	**10±6**	92	2.4
Hemopexin (HPX)	50-80 [6]	3.05E+10	**56±17**	29	0.7
Alpha-1-antichymotrypsin (SERPINA3)	40-70 [7]	1.05E+10	143±67	-	-

Figure S1. Depletion of the proteins considered in this study in HSA-depleted fraction. Based on the 4th column of Table S1. The red points beneath the red line are considered as having significantly lower levels of the ratio, thus significantly depleted.

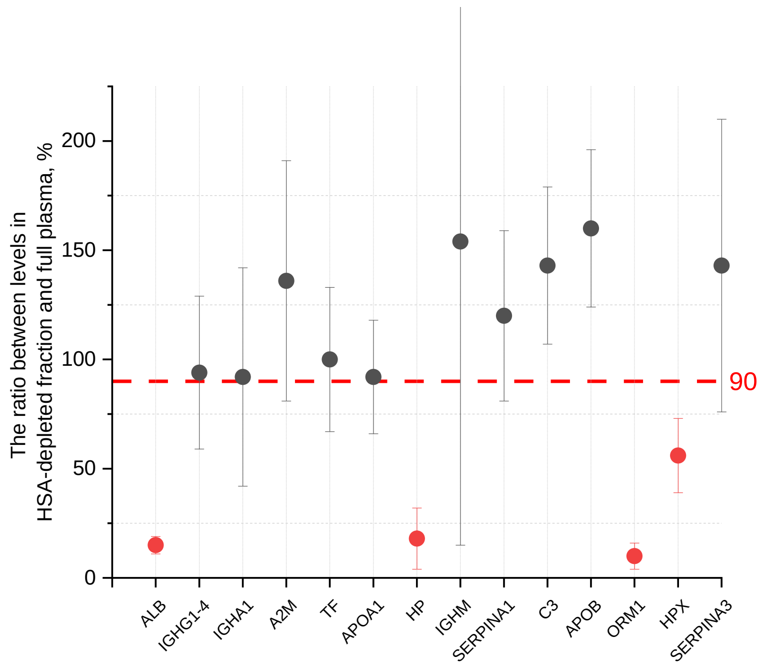

Figure S2. FTIR absorption spectra of the twelve proteins analyzed in the study, measured at the same concentration (5 mg/mL).

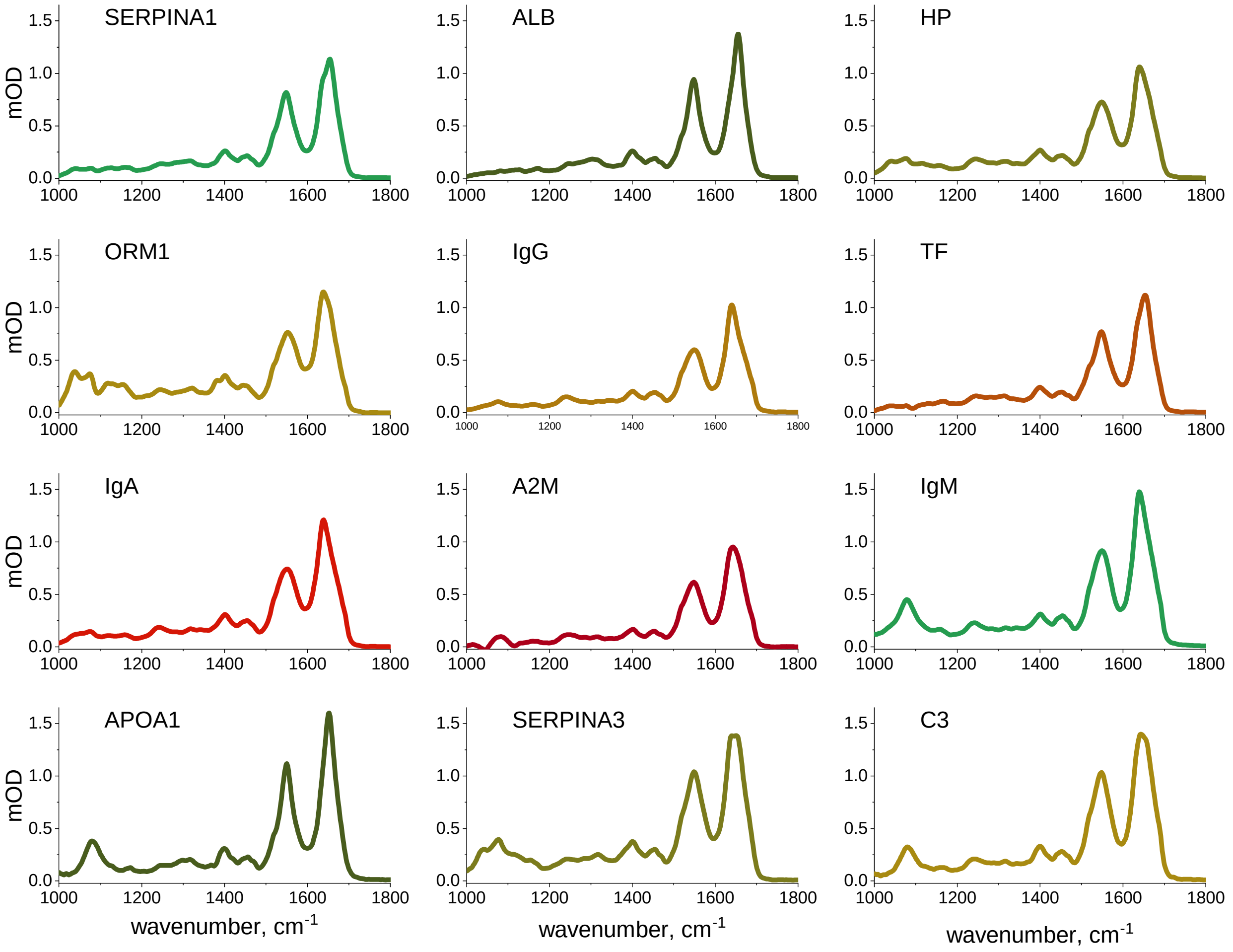

Figure S3. Agreement between the model and the experiment depending on the number of proteins taken into account and upon addition of the whole metabolite fraction to the model. The error bars show the standard deviation for all 148 samples.

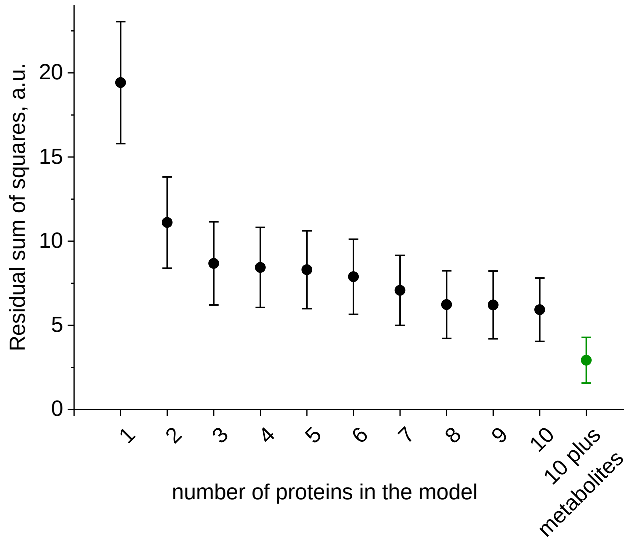

Table S2. Breakdown of the participants of the clinical study.

	Lung cancer patients (N=55)		Reference individuals (N=93)		
	**Adeno carcinoma**	**Squamous cell carcinoma**	**Chronic obstructive pulmonary disease (COPD) patients**	**Lung hamartoma patients**	**Non-symptomatic, healthy individuals**
**Number**	21	34	26	36	31
**Average age**	62.7	63.7	64.2	64.0	61.4
**% female**	43	38	35	42	42
**% active smokers**	43	52	19	47	42
**% ex-smokers**	24	45	77	39	39
**% TNM stage II**	29	47	n/a	n/a	n/a
**% TNM stage III**	71	53	n/a	n/a	n/a

Table S3. Infrared molecular fingerprints of blood serum of lung cancer patients and the reference cohort are significantly different, as shown in the left column for a set of wavenumbers where the p-values are the lowest. The IMFs of HSA-depleted and HSA also provide spectral features with low p-values. Finally, the lowest attenable p-value for metabolite fraction is shown for comparison in the right column. It demonstrates that there is little difference between the IMFs of the metabolite fractions of lung cancer patients and reference cohort.

Crude serum		***HSA-enriched fraction***		***HSA-depleted fraction***		***Metabolite-fraction***	
Wavenumber, cm^{-1}	*p-value*	*Wavenumber, cm^{-1}*	*p-value*	*Wavenumber, cm^{-1}*	*p-value*	*Wavenumber, cm^{-1}*	*p-value*
1070	10^{-6}	1025-1160	$<10^{-6}$				
1227-1256	$<10^{-9}$	1216-1269	$<10^{-7}$			1250	$1*10^{-2}$
1402	$3*10^{-11}$	1378	$2*10^{-8}$				
1516	$2*10^{-11}$	1432	$4*10^{-8}$				
1567	$1*10^{-11}$	1577	$3*10^{-8}$	1578	$7*10^{-8}$		
1650-1673	$<10^{-9}$	1651	$7*10^{-9}$				
				1733	$3*10^{-6}$		
				2836-2966	$<10^{-4}$		

Table S4. List of proteins that were found to differ most significantly in the comparison between the lung cancer patients and the reference cohort (p<0.0005) in the proteomic mass-spec analysis. The proteins used for modeling of differential fingerprints are highlighted in bold.

**Protein**	**p-value**	**change in lung cancer patients *vs* controls, %**	**mean AUC when only this protein is used for classification**
complement component C9 (C9)	7E-09	55	0.81
**haptoglobin (HP)**	2E-08	62	0.82
kallistatin (SERPINA4)	6E-08	-23	0.78
**alpha-1-antitrypsin (SERPINA1)**	6E-07	29	0.75
gelsolin (GSN)	2E-06	-18	0.78
leucine-rich alpha-2-glycoprotein (LRG1)	3E-06	67	0.75
**alpha-1-antichymotrypsin (SERPINA3)**	3E-06	46	0.76
serum amyloid P-component (APCS)	4E-06	25	0.72
plasma serine protease inhibitor (SERPINA5)	4E-06	-21	0.76
serum amyloid A-2-4 proteins (SAA2-SAA4; SAA4)	2E-05	36	0.70
complement factor B (CFB)	2E-05	19	0.73
ceruloplasmin (CP)	3E-05	20	0.69
**alpha-1-acid glycoprotein 1 (ORM1)**	3E-05	91	0.81
transthyretin (TTR)	6E-05	-24	0.76
N-acetylmuramoyl-L-alanine amidase (PGLYRP2)	9E-05	-15	0.75
apolipoprotein C-III (APOC3)	3E-04	-21	0.71
serum-derived hyaluronan-associated protein (ITIH3)	3E-04	26	0.69
lipopolysaccharide-binding protein (LBP)	4E-04	73	0.75
antithrombin-III (SERPINC1)	5E-04	-16	0.71

Table S5. List of proteins that differ most significantly – in terms of absolute change of their concentration – in the comparison between the lung cancer patients and the reference cohort, based on the proteomic analysis.

number	Protein	Change in lung cancer, mg/dL	Change in lung cancer, %
1	human serum albumin (ALB)	-384	-9
2	haptoglobin (HP)	108	62
3	alpha-1-acid glycoprotein 1 (ORM1)	93	91
4	alpha-1-antitrypsin (SERPINA1)	43	29
5	transferrin (TF)	-29	-11
6	immunoglobulin G (IGHG1, IGHG2, IGHG3, IGHG4)	27	3
7	alpha-1-antichymotrypsin (SERPINA3)	25	46
8	immunoglobulin A (IGHA1)	24	7
9	apolipoprotein A1 (APOA1)	-23	-13
10	alpha-2-macroglobilin (A2M)	-22	-7
11	complement component C3 (C3)	12	9
12	apolipoprotein B (APOB)	-10	-9
13	hemopexin (HPX)	8	13
…	…	…	…
20	immunoglobulin M (IGHM)	-2	-1

Figure S4. Average IMF of HSA-enriched fractions of 148 human blood sera, each modelled as a sum of contributions of 3 proteins compared to the average experimentally measured IMF of HSA-enriched fraction.

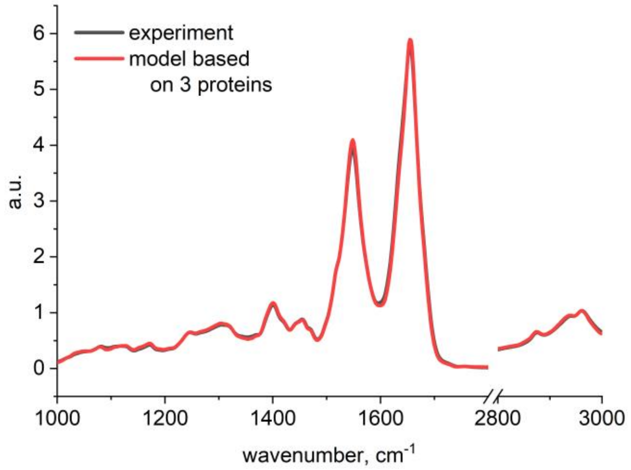

Table S6. List of proteins that together, when combined, provide the binary classification between lung cancer and the control condition with AUC value of 0.87±0.1, which is the highest value achieved using the proteomic data set combined with forward feature selection based on the SVM-classification performance.

Protein
haptoglobin (HP)
alpha-1-acid glycoprotein 1 (ORM1)
gelsolin (GSN)
apolipoprotein A1 (APOA1)
apolipoprotein A-IV (APOA4)
serum paraoxonase/arylesterase 1 (PON1)
complement component C9 (C9)

Materials and methods

1. Chemicals and reagents.

Methanol and ethanol of HPLC-grade, sodium chloride and proteins at highest available purity rate were purchased from Sigma Aldrich GmbH (Taufkirchen, Germany). The proteins that were purchased as powder were diluted in 20mM PBS buffer (Sigma Aldrich GmbH). If traces of additional salts were present in the protein solution, the buffer was exchanged to 20 mM PBS using Nanosep Omega centrifugal filters with 3 kD cutoff (VWR, Germany).

2. Clinical study participants.

We performed a clinical study on lung cancer, including subjects with benign conditions and non-symptomatic healthy volunteers as reference. The following clinical centers were involved in subject recruitment and sample collection: Department of Internal Medicine V for Pneumology, Ludwig-Maximillian-University (LMU) of Munich; Urology Clinic, LMU; Department of Obstetrics and Gynaecology, LMU; Breast Cancer and Comprehensive Cancer Centre Munich (CCLMU), LMU; Asklepios clinic, Gauting; Comprehensive Pneumology Centre (CPC), Munich, all located in Germany. All participants signed written informed consent form for the study under research Study Protocol # 17-182 or # 17-141, both approved by the Ethics Committee of the Ludwig-Maximillian-University (LMU) of Munich and performed in compliance with all relevant ethical regulations. Analyses focus on subjects with clinically confirmed carcinoma of lung at the TNM clinical stages II and III, with no metastases, prior to any cancer-related therapy, and without any other cancer occurrence. Healthy references were non-symptomatic individuals, without any history of cancer, not suffering from any cancer-related disease nor being under any medical treatment. Lung cancer cases were compared to matched individuals from the following groups: Chronic obstructive pulmonary disease (COPD), pulmonary hamartoma and non-symptomatic healthy individuals matched

for gender, age and smoking status. Full breakdown of all participants is listed in SI Appendix Table S2.

3. Blood sample collection and preparation

Blood samples were collected, processed and stored using previously defined standard operating procedures: Blood draws were all performed using Safety-Multifly needles of 21G (Sarstedt AG & Co KG, Germany) into 4.9 ml or 7.5 ml serum tubes, centrifuged at 2.000 g for 10 minutes at 20 °C, aliquoted and frozen at -80°C within 5 hours from the time of sampling. All samples were thawed, further aliquoted for measurement and re-frozen at -80°C to ensure a constant number of freeze-thaw cycles before analysis. Before any measurement, the aliquots were thawed at room temperature, shaken for 20 seconds, and spun down again.

4. Fourier-transform infrared spectroscopy measurements

Measurements of liquid biofluids, their fractions and single proteins were all performed in hydrated, fluid state using an automated FTIR device MIRA-Analyzer (Micro-biolytics GmbH, Germany) with a flow-through transmission cuvette (CaF_2 with 8 µm path length), as demonstrated previously.[8] The spectra were acquired with a resolution of 4 cm^{-1} and an averaging time of 45 s. After sample exchange a water reference spectrum was measured to reconstruct the infrared absorption spectra. Samples were measured in a random order to avoid systematic effects during data evaluation. To measure and track experimental errors during the measurement campaign, quality control samples from pooled human serum (BioWest, Nuaillé, France) were measured after each 5 samples.

The pre-processing of the experimental spectra was performed using home-built software and relies on the previous work [9,10]. The spectra were obtained in the range from 930 cm^{-1} to 3050 cm^{-1} and truncated to 1000-3000 cm^{-1}. Baseline correction was introduced to account for the water substituted by the blood constituents in the sample compared to the pure-water reference. In particular, water absorption spectrum was added to the sample spectrum with a coefficient optimized such that the first derivative of the signal at 1800-2200 cm^{-1} (2200-2400 cm^{-1} for the metabolite fraction) is minimal [10]. Subsequently, the minimum of the absorption in this region is subtracted from the spectrum (“offset correction”). Finally, vector-normalization was used to reduce experimental noise [11].

The absorption spectra of single proteins were measured at 1 to 5 mg/mL concentration depending on the sample availability. The spectrum of PBS buffer was subsequently measured and subtracted from the protein spectrum prior to further pre-processing. When the buffer was exchanged using centrifugal filters, which implies sample loss, the resulting protein concentration was determined using BCA Protein Assay.

5. UPLC-MS proteomics measurements

Sample preparation was carried out according to our Plasma Proteome Profiling pipeline [12,13], employing an automated setup on an Agilent Bravo Liquid Handling Platform. In brief, 1 µl of each plasma sample or input from the HSA depletion method was aliquoted into 24 µl of lysis buffer (P.O. 00001, PreOmics GmbH) in a 96 well plate (Eppendorf twin.tec PCR LoBind). Reduction of disulfide bridges, cysteine alkylation, and protein denaturation was performed at 95°C for 10 min. [14] Trypsin and LysC were added to the mixture after a 5-min cooling step at room temperature, at a ratio of 1:100 micrograms of enzyme to micrograms of protein. Digestion was performed at 37°C for 1 h. An amount of 0.5 µg of peptides was loaded to Evotips (Evosep, Odensee, Denmark) following the manufacturer protocol.

Samples were measured using LC-MS instrumentation consisting of an Evosep One (Evosep, Odensee, Denmark) [15], which was coupled to a Q Exactive HF-X Orbitrap (Thermo Fisher Scientific) using a nano-electrospray ion source (Thermo Fisher Scientific). Purified peptides were separated on 15-cm HPLC columns [ID: 150 μm; in-house packed into the tip with ReproSil-Pur C18-AQ 1.9 μm resin (Dr. Maisch GmbH)]. For each LC-MS/MS analysis, about 0.5 μg peptides were used for 21 min run. Column temperature was kept at 60°C by an in-house-developed oven containing a Peltier element, and parameters were monitored in real time by the SprayQC software [16]. MS data were acquired with a Top12 data-dependent MS/MS scan method. Target values for the full-scan MS spectra were 3×10^6 charges in the 300–1,650 m/z range with a maximum injection time of 50 ms and a resolution of 60,000 at m/z 200. Fragmentation of precursor ions was performed by higher-energy C-trap dissociation (HCD) with a normalized collision energy of 27 eV. MS/MS scans were performed at a resolution of 15,000 at m/z 200 with an ion target value of 5×10^4 and a maximum injection time of 25 ms. Dynamic exclusion was set to 15 s to avoid repeated sequencing of identical peptides.

MS raw files were analyzed by MaxQuant software, version 1.6.3.3 [17], and peptide lists were searched against the human UniProt FASTA database. A contaminant database generated by the Andromeda search engine [18] was configured with cysteine carbamidomethylation as a fixed modification and N-terminal acetylation and methionine oxidation as variable modifications. We set the false discovery rate (FDR) to 0.01 for protein and peptide levels with a minimum length of 7 amino acids for peptides, and the FDR was determined by searching a reverse database. Enzyme specificity was set at C-terminal to arginine and lysine as expected using trypsin and LysC as proteases. A maximum of two missed cleavages were allowed. Peptide identification was performed with an initial precursor mass deviation up to 7 ppm and a fragment mass deviation of 20 ppm. All proteins and peptides matching to the reversed database were filtered out. Label-free protein quantitation (LFQ) was performed with a minimum ratio count of 2 [19].

6. Fractionation of blood serum

All the samples and all reagents were kept at 4^0C during the process. The samples were processed in batches of 24, including 4 quality control samples per each batch (see above). Two-step fractionation of liquid biofluids has been performed. The goal of the first step is to separate most of the proteins from the suman serum albumin (HSA) and follows the previously proposed [20]. To that end, 0.1M NaCl and 42% of ethanol have been added to the samples. They were vortexed for 1 hour, then centrifuged for 20 minutes at 16000 rcf, so that a pellet containing most of the proteins (HSA-depleted fraction) is formed. The supernatant that contains HSA and metabolites was transferred to a new tube, while the pellet was re-dissolved in water *via* vortexing for 1.5 hours. A small pellet was left in the tube. We have shown that if the HSA-depleted protein pellet is vortexed for longer, the left-over pellet is reduced, but the FTIR spectra of the supernatant are identical to those obtained after 1.5 hours. The HSA-depleted fraction has been transferred to a new tube and placed into the vacuum concentrator (Concentrator plus, Eppendorf GmbH, Germany) for 3 hours. To avoid clogging of the automated measurement system, the HSA-depleted fraction has been centrifuged for 15 minutes at 15000 rcf and frozen at -80^0C until further use.

To separate HSA-enriched protein fraction from metabolites, we added 59% of pre-cooled methanol, vortexed the samples for 1 minute and centrifuged for 15 minutes at 15000 rcf, so that a pellet containing HSA and other proteins was formed [21]. The supernatant was transferred to a new tube and fully dried in the concentrator in 3 hours. The metabolites were then re-dissolved in water *via* vortexing for 2 minutes and frozen at -80^0C until further use.

The HSA-enriched pellet was fully re-dissolved in water *via* vortexing for 2 minutes and placed into the vacuum concentrator for 3 hours. The resulting HSA-enriched protein fraction was frozen at -80^{0}C until further use. The total time required to process a sample batch was 8 hours.

7. Classification models

The data analysis was performed using the *Scikit-Learn* [22] (v. 0.23.2) module in Python (v.3.7.6). We trained classification models based on linear support vector machines (SVM) algorithm – as implemented in the *LinearSVC* class with default parameters. We evaluated the model using stratified 10-fold cross validation, repeated 10-times with different randomization in each repetition. For the visualization of the model performance, we use the notion of the receiver operating characteristic (ROC) curve. As an overall metric for model performance, we use the area under the ROC curve (AUC). For the evaluation of the mass-spectrometry data, we performed ranking of individual proteins using forward feature selection based on the SVM-classification performance.

Supplementary references

[1] R. F. Ritchie, G. E. Palomaki, L. M. Neveux, O. Navolotskaia, T. B. Ledue, W. Y. Craig, *J. Clin. Lab. Anal.* **1999**, *13*, 273–279.

[2] A. Kratz, M. Ferraro, P. M. Sluss, K. B. Lewandrowski, *N. Engl. J. Med.* **2004**, *351*, 1548–1563.

[3] R. F. Ritchie, G. E. Palomaki, L. M. Neveux, O. Navolotskaia, *J. Clin. Lab. Anal.* **1998**, *12*, 371–377.

[4] J. Housley, *J. Clin. Pathol.* **1968**, *21*, 27–31.

[5] R. F. Ritchie, G. E. Palomaki, L. M. Neveux, O. Navolotskaia, *J. Clin. Lab. Anal.* **2000**, *14*, 265–270.

[6] U. Muller-Eberhard, J. Javid, H. H. Liem, A. Hanstein, M. Hanna, *Blood* **1968**, *32*, 811–815.

[7] F. Licastro, E. Masliah, S. Pedrini, L. J. Thal, *Dement. Geriatr. Cogn. Disord.* **2000**, *11*, 25–28.

[8] M. Huber, K. V. Kepesidis, L. Voronina, M. Božić, M. Trubetskov, N. Harbeck, F. Krausz, M. Žigman, *Nat. Commun.* **2021**, *12*, 1511.

[9] H. J. Butler, B. R. Smith, R. Fritzsch, P. Radhakrishnan, D. Palmer, M. J. Baker, *Analyst* **2018**, 6121–6134.

[10] P. Lasch, *Chemom. Intell. Lab. Syst.* **2012**, *117*, 100–114.

[11] R. Aruga, *Talanta* **1998**, *47*, 1053–1061.

[12] P. E. Geyer, N. A. Kulak, G. Pichler, L. M. Holdt, D. Teupser, M. Mann Correspondence, M. Mann, *Cell Syst.* **2016**, *2*, 185–195.

[13] P. E. Geyer, N. J. W. Albrechtsen, S. Tyanova, N. Grassl, E. W. Iepsen, J. Lundgren, S. Madsbad, J. J. Holst, S. S. Torekov, M. Mann, *Mol. Syst. Biol.* **2016**, *12*, 901.

[14] N. A. Kulak, G. Pichler, I. Paron, N. Nagaraj, M. Mann, *Nat. Methods* **2014**, *11*, 319–324.

[15] N. Bache, P. E. Geyer, D. B. Bekker-Jensen, O. Hoerning, L. Falkenby, P. V. Treit, S. Doll, I. Paron, F. Meier, J. V. Olsen, O. Vorm, M. Mann, *bioRxiv* **2018**, 1–20.

[16] R. A. Scheltema, M. Mann, *J. Proteome Res.* **2012**, *11*, 3458–3466.

[17] J. Cox, M. Mann, *Nat. Biotechnol.* **2008**, *26*, 1367–1372.

[18] J. Cox, N. Neuhauser, A. Michalski, R. A. Scheltema, J. V Olsen, M. Mann, *J. Proteome Res.* **2011**, *10*, 1794–1805.

[19] J. Cox, M. Y. Hein, C. A. Luber, I. Paron, N. Nagaraj, M. Mann, *Mol. Cell. Proteomics* **2014**, *13*, 2513–2526.

[20] D. A. Colantonio, C. Dunkinson, D. E. Bovenkamp, J. E. Van Eyk, *Proteomics* **2005**, *5*, 3831–3835.

[21] D. Vuckovic, *Anal. Bioanal. Chem.* **2012**, *403*, 1523–1548.

[22] F. Pedregosa, G. Varoquaux, A. Gramfort, V. Michel, B. Thirion, O. Grisel, M. Blondel, P. Prettenhofer, R. Weiss, V. Dubourg, J. Vanderplas, A. Passos, D. Cournapeau, M. Brucher, M. Perrot, E. Duchesnay, *J. Mach. Learn. Res.* **2011**, *12*, 2825–2830.
